# Antibiogram pattern of Enterococcus species among urinary tract-infected patients visiting tertiary care hospital in Karnataka, India

**DOI:** 10.1017/ash.2024.419

**Published:** 2024-11-14

**Authors:** Sujatha Bhat, Dushan Uvindu Gunawardana, Delisha Kaur Boparai, Tharinya Kasundie Bamunusinghe, Kavin Krishanth, Aarabi Premakrishna, Nagalakshmi Narasimhaswamy

**Affiliations:** 1 Division of Microbiology, Department of Basic Medical Sciences, Manipal Academy of Higher Education, Manipal, Karnataka, India; 2 Melaka Manipal Medical College (Manipal Campus), Manipal Academy of Higher Education, Manipal, Karnataka, India

## Abstract

**Background:**

During the past several decades, enterococci are emerging as an important cause of healthcare-associated infections. They have developed resistance to various antimicrobials previously used for the treatment of urinary tract infections (UTIs). This study was aimed to determine the prevalence of *Enterococcus species* among urinary tract-infected patients in a tertiary care hospital, in Karnataka, India.

**Material and Methods:**

We have analyzed 4341 culture-positive urine samples received by microbiology laboratory during the year 2021. The bacterial identification was done by matrix-assisted laser desorption ionization-time of flight mass spectrometry. The antibiotic sensitivity was tested by automated VITEK-2® COMPACT (bioMérieux) system.

**Results:**

Among 4341 culture-positive samples, Enterococcal species were isolated from 159 samples. A total of 64.7% of the isolates were identified as *Enterococcus faecalis* and 28.3% of the strains as *Enterococcus faecium*. All the enterococci were sensitive to linezolid, teicoplanin, and vancomycin, whereas 59.1%, 30.9%, and 23.3% of the strains exhibited resistance to high-level gentamicin, benzylpenicillin, and nitrofurantoin, respectively. 33.67 % of the isolates were identified as multidrug-resistant (MDR) strains as they exhibited resistance to high-level gentamicin, benzylpenicillin, and nitrofurantoin.

**Conclusion:**

Our study shows the prevalence of *Enterococcus faecalis* and high-level gentamicin-resistant enterococcal strains. The MDR pattern of enterococci requires careful consideration of antimicrobial therapy to treat UTIs. The reserved drugs such as linezolid, vancomycin, and teicoplanin should be cautiously used for the treatment of enterococcal UTI.

## Introduction

Urinary tract infections (UTIs) are a group of common diseases that occur primarily when the normal enteric flora ascends in the urinary bladder through urethra.^1^ *Escherichia coli*, *Proteus species*, *Enterobacter species*, *Staphylococcus saprophyticus*, *Staphylococcus aureus*, *Enterococcus species*, and certain fungi such as *Candida species* are the frequent causative agents of UTIs.^2,3^ During the past few decades, Enterococcus species have emerged as an important nosocomial uropathogen.^4–7^ Enterococcus species are the second most common nosocomial pathogen after *Staphylococcus aureus* and are responsible for 15%–20% of hospital-acquired infections.^8–10^ The pathogenicity of Enterococcus species is due to the active secretion of several virulence factors like cytolysins (hemolysin/cytolysin), gelatinase, secreted antigen A, and various cell surface factors such as pili and cell surface proteins. These factors are responsible for the environmental survival of this organism by rendering bacterial adhesion to a variety of surfaces, biofilm formation, and inhibition of complement-mediated immunity.^11^ Though there are more than 58 *Enterococcus species*,^8^ most UTIs were found to be caused by *E. faecalis* and *E. faecium.* In addition to UTIs, Enterococcus species are also implicated in causing endocarditis, bacteremia, meningitis, intra-abdominal infections, septic arthritis, osteomyelitis, pneumonia, and wound infections.^8,9^

Enterococcus species have evolved and developed resistance toward various antimicrobials previously used for the treatment of UTIs. The rate of development of resistance toward antibiotics by these species is rapidly increasing and multidrug-resistant (MDR) enterococci have been shown to exhibit a wide range of antibiotic resistance mechanisms. Modification of drug targets, overexpression of efflux pumps, decreased drug uptake, and inactivation of drugs are some of the mechanisms.^12^ The acquisition of resistance to β-lactam drugs, aminoglycosides, high- and low-level vancomycin, etc, are posing a greater difficulty in treating the clinical conditions caused by this organism. In view of this, we have conducted a study to identify the antibiotic susceptibility pattern of Enterococcus species by analyzing culture and sensitivity reports of UTI patients visited a tertiary care hospital, Karnataka, India. The study results could be used to understand the epidemiological aspects of antimicrobial-resistant strains of Enterococcus species causing UTIs.

## Materials and methods

This retrospective study was conducted between January 2021 and December 2021 consisting of urine samples of 8845 UTI patients who have visiting to a tertiary care hospital, Karnataka, India, for treatment. Urine specimens positive for Enterococcus species identified from laboratory registers of the microbiology department were included in the study. The Institutional Ethical Committee approval was taken to conduct this study (IEC 2: 14/2022).

Mid-stream urine or catheterized urine specimen obtained was immediately processed in the microbiology laboratory. Using a calibrated wire loop, the urine samples were cultured onto 5% sheep blood agar and MacConkey agar and incubated at 37°C. A semi-quantitative urine culture technique was followed to calculate the growth. The bacterial phenotypic identification was done by matrix-assisted laser desorption ionization-time of flight mass spectrometry (MALDI-TOF), and automated VITEK-2® COMPACT (bioMérieux) system was used to determine the antibiotic sensitivity pattern of Enterococci. We have included high-level gentamicin, benzylpenicillin, nitrofurantoin, linezolid, vancomycin, and teicoplanin for antibiotic susceptibility testing. *Enterococcus faecalis* ATCC 29212 strain was used as a quality control. The data from the laboratory records were entered into the Microsoft Excel sheet, and frequency of occurrence of Enterococcus species and their antibiogram pattern were analyzed and represented in percentage.

## Results

**Figure 1.**
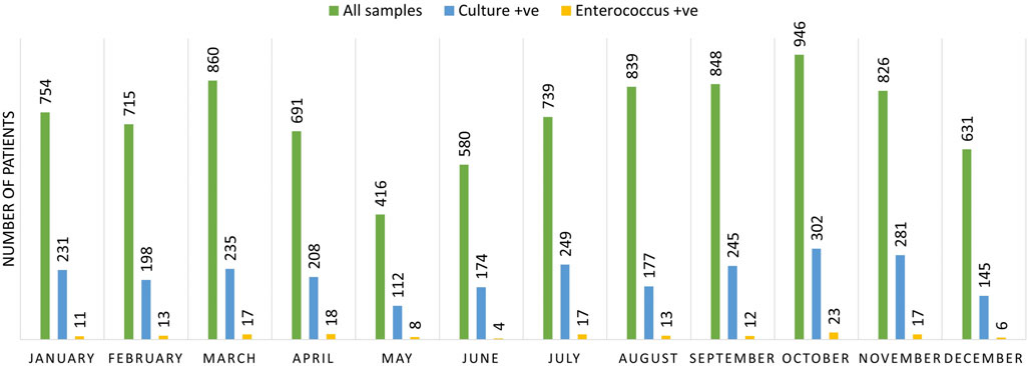
Prevalence of Enterococcus species among UTI patients in 2021 (month-wise distribution).

Figure 1 shows the cumulative sum of samples collected throughout the year 2021 on a monthly basis, along with the total culture-positive samples of Enterococcal isolates. From our study, we have observed that the relative occurrence of Enterococcus strains isolated were 3.66%. A total of 159 strains of Enterococcus species were isolated from a total of 4341 culture-positive samples. Among 159 Enterococcus species isolated, 64.7% were identified as *E. faecalis* and 28.3% as *E. faecium*. Species identification of Enterococci was not determined for the remaining 7% of the isolates. It has been observed that 43.39% of *Enterococcus species* were isolated from the patients visited to the outpatient department and 56.60% were from hospitalized patients. We have also observed that 56.6% of females and 43.4% of males had significant growth of Enterococcus species in their urine samples. This indicates the higher rate of incidence of enterococcal infection in females than in males in our study population. Enterococcal infection was most frequently found in the adult group (50.27% in the age group of 18–60 years) followed by elderly patients (42.13%) and children (7.6%).

**Figure 2.**
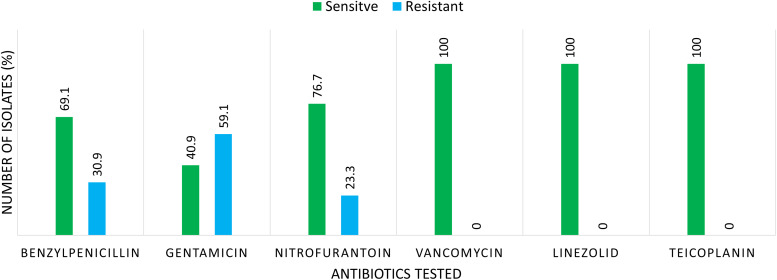
Antibiotic sensitivity pattern of Enterococcus species isolated from urine samples.

The antibiotic sensitivity of the enterococci revealed that 38.36% of the isolates were sensitive to all the antibiotics tested, whereas 61.64% of the isolates showed antibiotic sensitivity pattern of various combinations. Figure 2 shows the resistance pattern of Enterococcus species to various antibiotics. It is clear from the figure that 59.1% the isolates were resistant to high-level gentamicin, followed by benzylpenicillin resistance (30.9%) and resistance to nitrofurantoin (23.3%). All the Enterococcus species showed absolute sensitivity (100%) to reserve drugs used (ie, linezolid, vancomycin, and teicoplanin).

**Figure 3.**
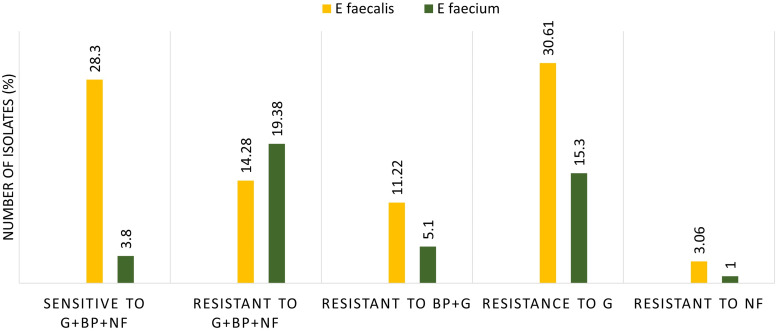
Antibiotic resistance pattern of Enterococcus species from urine samples (antibiotics: G: gentamicin, BP: benzylpenicillin, and NF: nitrofurantoin).

Figure 3 shows the details of MDR strains of Enterococcus species. A MDR bacteria refers to an isolate which is resistant to at least one antibiotic in three or more drug classes. In our study group, 33.67% of the Enterococcus isolates found to be MDR (ie, resistance high-level gentamicin, benzylpenicillin, and nitrofurantoin). Among these MDR isolates, 14.28% of them were *E. faecalis* and 19.38% of them found to be *E. faecium*. When we compared the MDR and sensitive strains of enterococci to the patient’s admission details, we observed that 78.8% of MDR strains (26 isolates from a total of 33 MDR isolates) were isolated from hospitalized patients.

## Discussion

Though Enterococcus species have been more considered as the common colonizer of the human intestinal tract than infectious agents, they are found to be most often associated with UTIs. Several factors such as age, gender, diabetes mellitus, pregnancy, and immunosuppression are associated with higher incidence of enterococcal infections of UTI.^13^ We have observed a higher incidence of enterococcal infections in females (56.6%) than in males. Similar findings were also observed by Naruka et al. (2019).^14^ Proximity of anus and urethra in females and the effect of hormones such as estrogen could be the reason for the higher incidence of enterococcal infection. Also, the highest proportion of isolates (42.13%) were observed in patients above 60 years of age. This may be due to urinary retention, immune weakness, and other co-morbid conditions, which are common in that age group. The age group ranging from 0 to 18 years contributed 7.6% of the total isolates and most of them being infants < 12 months in age. Infection in this age group may be due to immature immune system. *Enterococcus faecalis* was the most frequent Enterococcus species found in the current study, comprising 64.7% of the total isolates. This finding was similar with many other studies.^15–17^

Enterococcus species exhibiting multiple-drug resistance are common these days. In general, among the Enterococcus species, *E. faecium* is inherently resistant to many antibiotics than *E. faecalis* and most isolates exhibit resistance to ampicillin, aminoglycosides, and vancomycin.^18–20^ Our study also revealed that *E. faecium* strains outnumbered *E. faecalis* strains in exhibiting multidrug resistance (resistance to high-level gentamicin, benzylpenicillin, and nitrofurantoin). In most cases, Enterococcus species causing UTIs are often acquired in hospital settings, and consequently, they acquire resistance to many antibiotics. In support of this, we found that most MDR strains (ie, 78.8%) were isolated from hospitalized patients.

We have observed that 16.3% of *Enterococcus species* exhibited resistance to both β-lactams and high-level gentamicin in our study. In clinical settings, enterococcal infections are often treated with a synergistic combination of cell wall-inhibiting agents (penicillin or a glycopeptide) and aminoglycoside. Though low-level intrinsic aminoglycoside resistance is common in enterococci, occurrence of increased rate of high-level resistance to aminoglycosides in enterococci is alarming. This problem is primarily due to acquisition of aminoglycoside-modifying enzymes, which eliminates the synergistic bacterial killing effect of aminoglycoside and β-lactam antibiotics.^21^ The three reserve drugs, that is, linezolid, vancomycin, and teicoplanin, showed absolute sensitivity against Enterococcus species throughout the study period. So, it is apparent that, though high-level aminoglycoside resistance toward gentamicin is increasing at an alarming rate, vancomycin-resistant enterococci (VRE) are not of much concern in this locality. However, countrywide information showed a notable rise in VRE prevalence. Smout et al.^22^ have studied the prevalence of enterococci in India by considering studies conducted across the country between 2000 and 2022. Out of six studies, the overall prevalence of VRE as an uropathogen was calculated to be 14.42% (133 VRE from 922 enterococci isolated). Also, the authors reported that, after considering 3683 Enterococci isolates from various clinical specimens, the overall prevalence of VRE in India was 12.4%. In comparison to our report, VRE prevalence is higher in other parts of India. So, a thorough knowledge of antibiotic sensitivity pattern of enterococci in an area is essential to formulate antibiotic policy.

Enterococcus species are implicated not only in UTIs, but a plethora of many other diseases like sepsis, meningitis, and more commonly endocarditis. Although this study was focused on Enterococcus species as an etiology for UTIs, the frequent occurrence of this organism, especially in healthcare settings, affects the course of all said clinical conditions in due time. Further, the emergence of MDR strains of this organism threatens and questions the prevailing protocols and the guidelines of hospitals throughout the world in treatment of such conditions. Therefore, comprehensive infection control programs that can minimize the spread of these resistant organisms are essential.

**Limitation of the study:** The present study has one major limitation. Since it is a retrospective study, molecular methods for the identification of virulent genes of enterococci, responsible for their antibiotic resistance, were not conducted.

## Conclusion

This study shows the prevalence of *E. faecalis* in our geographical region. The occurrence of MDR strains, especially high-level gentamicin-resistant enterococcal stains, are posing a greater challenge to the clinicians to prevent their spread. Hence, careful consideration of antimicrobial therapy is needed to control the infections caused by these strains. We can also conclude from our study that, though high-level gentamicin-resistant enterococcal stains are prevailing, yet resistance to vancomycin is absent in this locality. Therefore, drugs such as linezolid, vancomycin, and teicoplanin can be effectively used in the treatment and management of enterococcal UTI.
